# Absence of lipopolysaccharide (LPS) expression in breast cancer cells

**DOI:** 10.1007/s13402-025-01071-8

**Published:** 2025-06-11

**Authors:** Noel F. C. C. de Miranda, Vincent T. H. B. M. Smit, Manon van der Ploeg, Jelle Wesseling, Jacques Neefjes

**Affiliations:** 1 Department of Pathology, Leiden University Medical Center, Leiden, The Netherlands; 2 Department of Pathology, The Netherlands Cancer Institute – Antoni van Leeuwenhoek, Amsterdam, The Netherlands; 3 Division of Molecular Pathology, The Netherlands Cancer Institute, Amsterdam, The Netherlands; 4 Department of Cell and Chemical Biology, ONCODE Institute, Leiden University Medical Center, Leiden, The Netherlands

**Keywords:** Lipopolysaccharide (LPS), Microbiome, Breast cancer, Macrophages, Immunohistochemistry

## Abstract

The relationship between bacterial activity and tumorigenesis has gained attention in recent years, complementing the well-established association between viruses and cancer. A recent study employed immunodetection of lipopolysaccharide (LPS) to demonstrate the presence of intracellular bacteria within cancer cells across various cancer types, including breast cancer. The authors proposed that these bacteria might play a role in tumor development. We sought to replicate these findings using the same experimental methods on an independent cohort of breast cancer cases. Our analysis of 129 samples revealed no evidence of LPS expression within cancer cells. Instead, LPS immunoreactivity was observed in ducts or immune cells, specifically macrophages, as expected. These discrepancies in LPS immunodetection warrant caution in interpreting the original findings, and further research is needed to clarify the potential role of intracellular bacteria in cancer development.

## Brief communication

While the etiological role of viruses in cancer has long been appreciated, bringing to light associations between bacterial activity and tumorigenesis has proven more challenging. Nevertheless, groundbreaking epidemiological and cell biological studies have, in recent years, connected particular bacterial pathogens such as *Salmonella typhi* and *Helicobacter pylori* to gallbladder carcinoma and gastric cancer, respectively [1, 2]. Furthermore, bacteria-derived genotoxic signatures have been observed in colorectal cancer [3, 4].

A previous report proposed that a substantial proportion of cancers harbor specific intracellular bacteria within the cancer cells themselves [5]. Bacterial 16 S ribosomal gene sequencing across hundreds of tumor samples revealed detectable bacterial DNA in 14% of melanomas and 63% of breast cancers. To assess bacterial localization, the authors performed immunohistochemical detection using an anti-lipopolysaccharide (LPS) antibody, which detected LPS in both cancer and immune cells. In addition, 16 S RNA in situ hybridization was conducted. The immunohistochemical approach, in particular, indicated that a substantial proportion of cancers—such as melanomas and breast cancers—exhibited LPS expression within cancer cells, which was interpreted as evidence of intracellular bacteria.

This observation was striking because LPS would be expected to trigger a robust immune response, potentially leading to a strong inflammatory response or even sepsis [6]. However, despite the abundant LPS immunodetection in many breast cancer tissues reported by Nejman et al. [5], breast cancer is generally not regarded as a particularly immunogenic tumor type [7]. Additionally, the reported subcellular localization of LPS is notable: the images presented by the authors suggest that LPS localizes to the nucleus, which is an unlikely location for intracellular bacteria [8].

These considerations prompted us to replicate the observations reported by Nejman et al. [5]. For this purpose, we assembled three independent cohorts. A commercial tissue microarray (BR10010f-BX), was purchased from US Biomax and included 50 cases of breast carcinoma with matched lymph node metastases. The second cohort consisted of a tissue microarray obtained from the Netherlands Cancer Institute, comprising 42 ductal and 15 lobular breast carcinomas. The third cohort included 22 biopsies from invasive breast cancers diagnosed at the Leiden University Medical Center. All retrospective analyses involving medical data and biospecimens were conducted in accordance with Dutch legislation and international ethical standards.

**Fig. 1:**
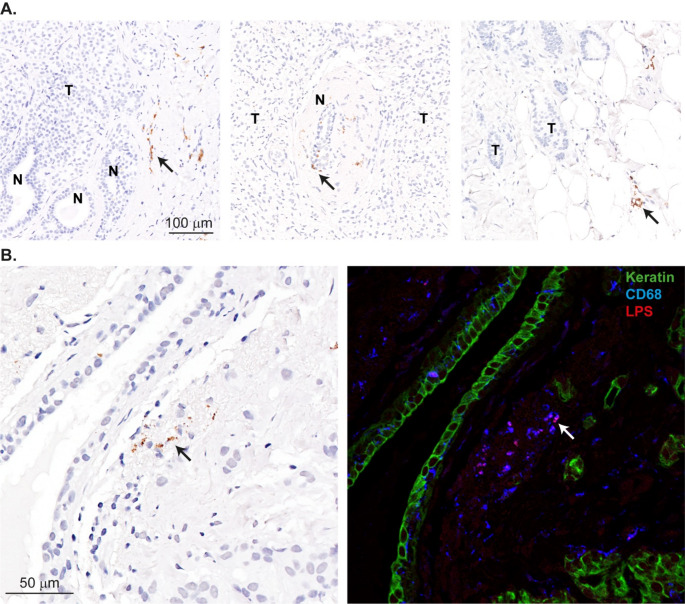
**A** - Representative examples of lipopolysaccharide (LPS) immunodetection (arrows) showing a typical granular pattern. LPS expression did not co-localize with tumor cells (T – tumor cells; N – normal breast ducts). **B** – Left: immunohistochemical detection of LPS in a breast cancer tissue section. Right: triple immunofluorescence of the next slice in this breast cancer tissue stained for anti-Keratin (green), anti-CD68 (blue) and anti-LPS (red) antibodies to show that LPS localized in CD68 positive cells. CD68 marks macrophages, supporting the detection of LPS inside macrophages. Bar indicates magnification

We assessed LPS expression using the same antibody clone employed by Nejman et al. (WN1 222-5, HycultBiotech) [5]. LPS immunoreactivity was observed in 12%, 5%, and 14% of samples in the three respective cohorts (totaling 129 breast cancer cases). Notably, LPS expression was never detected in cancer cells themselves, but rather in stromal regions or within ducts (Fig. 1A).

Since macrophages play a key role in defending tissues against bacterial invasion, we investigated whether LPS immunodetection in stromal cells originated from macrophages. To test this, we performed multiplex immunofluorescence using an anti-LPS antibody (1:200 dilution, clone WN1 222-5, HycultBiotech) and an anti-CD68 antibody (1:200 dilution, clone D4B9C, Cell Signaling), detected with Goat anti-Mouse IgG-Alexa Fluor 546 and Goat anti-Rabbit IgG-Alexa Fluor 647 (Invitrogen), respectively. Cancer cells were also identified using an anti-pan-Keratin antibody directly conjugated with Alexa Fluor 488. As hypothesized, our results confirmed that the stromal LPS signal originated from macrophages (Fig. 1B).

Previous efforts to detect LPS in gallbladder carcinomas - tumors strongly associated with chronic *Salmonella typhi* infection – also failed to show LPS expression, despite the presence of *S. typhi*-derived DNA in the same samples [1]. Therefore, we do not dispute the presence of bacterial DNA in tumor tissues, as reported by Nejman and colleagues. However, we do question the immunohistochemical findings that support the claim that intracellular bacteria are present within cancer cells [5]. The LPS immunodetection reported in that study, which suggested the widespread presence of LPS in human cancer tissues, particularly, in the nucleus of cancer cells, could not be reproduced in any of the 129 breast cancer samples we analyzed. However, in our cohort, the localization of LPS was consistent with its known biology, appearing in the lumen of breast ducts and within macrophages – sites where bacterial material would be expected. The divergent results may be due to inconsistencies between antibody batches or other methodological issues, such as bacterial contamination of the tissues.

The failure to replicate the detection of LPS in breast cancer cells contributes to the ongoing discussion surrounding microbiome-focused approaches that claim to find bacterial components within human cancers. In particular, recent bioinformatic studies that report microbial sequences in cancer genomic [9, 10] have been criticized for methodological flaws and potential misinterpretation of results [11–13]. This discussion emphasizes the need for careful application of bioinformatic analyses, ideally supported by independent biological validation—although such experiments must also undergo rigorous scrutiny, as illustrated by the lack of reproducibility in detecting LPS within cancer cells.

## Data Availability

No datasets were generated or analysed during the current study.
